# 3D Human Pose Estimation from Monocular Video Sequences in Underwater Scenarios

**DOI:** 10.3390/s26154738

**Published:** 2026-07-26

**Authors:** Shuwen Liang, Hailong Liu, Ping Liu, Dong Zhang, Rong Yu, Xiaowei Zhou, Zhize Zhou

**Affiliations:** 1 Sports Biomechanics Center, Institute of Artificial Intelligence in Sports, Capital University of Physical Education and Sports, Beijing 100191, China; suweenliang@163.com (S.L.); zhangdong@cupes.edu.cn (D.Z.); 2 State Key Laboratory of CAD&CG, Zhejiang University, Hangzhou 310058, China; hailong@zju.edu.cn (H.L.); xwzhou@zju.edu.cn (X.Z.); 3 Department of Leisure and Social Sports, Capital University of Physical Education and Sports, Beijing 100191, China; yurong@cupes.edu.cn

**Keywords:** underwater, human pose estimation, synthetic dataset

## Abstract

This paper presents a novel approach for estimating 3D human pose from monocular video sequences in underwater scenarios, tackling the unique challenges posed by water refraction, body occlusion, low image quality and illumination distortion in underwater environments. Leveraging both 2D keypoint extraction and parametric model estimation, our method operates in a two-stage framework including preprocessing and optimization. In the preprocessing stage, a Part Attention Regressor (PARE) is adopted to dynamically estimate SMPL human body parameters, particularly adept at handling occlusions common in underwater scenarios. Additionally, a 2D keypoint detector, employing YOLO for bounding box detection and HRNet for keypoint regression, enhances feature extraction despite underwater image challenges. In the optimization stage, we propose an underwater variational autoencoder (UW-VAE), which adopts a data-driven strategy to learn the biomechanical prior distribution of underwater human poses and implicitly correct unreasonable pose parameters caused by refraction and occlusion. The optimization process incorporates constraints aligning final SMPL models with detected 2D keypoints, minimizing disparity between adjusted and original SMPL models, and ensuring temporal consistency. Furthermore, to address the scarcity of annotated underwater datasets, we build a full pipeline to generate synthetic underwater datasets with complete annotations based on UW-VAE. Experimental results on the SwimXYZ synthetic dataset show that our method achieves 51.60% PCK@0.2 and 80.13% PCK@0.5, outperforming state-of-the-art land-based methods across most stroke categories. Validation on real-world underwater swimming datasets demonstrates improved 2D keypoint accuracy after synthetic-data fine-tuning, which provides a new solution for 3D human motion analysis in underwater sports, biomechanical research and swimming training.

## 1. Introduction

3D human pose estimation in underwater scenarios is a core technology with broad applications, including swimming technique optimization, athlete training evaluation, underwater biomechanics research, and drowning prevention. The perception of human movement and posture in underwater settings has significant implications across various domains, such as aquatic sports [1,2], underwater robotics [3], marine biology [4], and safety applications [5].

Compared with land-based motion capture, underwater motion analysis faces distinct and compounded challenges. The aquatic environment introduces severe visual degradations, including light attenuation, scattering, and refraction at the water–air interface, which fundamentally alter the appearance of human subjects [6,7]. Moreover, traditional multi-camera motion capture systems are difficult to deploy underwater due to calibration complexity and hardware constraints, while manual annotation of underwater videos remains time-consuming, labor-intensive, and prone to subjective errors. These factors collectively limit the scalability and accuracy of existing pose estimation pipelines in aquatic settings.

Recent years have witnessed substantial progress in monocular 3D human pose and shape (HPS) estimation for terrestrial scenes [8,9,10], driven by parametric body models such as SMPL (Skinned Multi-Person Linear) [11] and powerful deep learning frameworks. However, these methods typically assume clear imaging conditions and abundant annotated training data—assumptions that are violated underwater. Meanwhile, existing underwater vision research has predominantly focused on 2D keypoint detection or object tracking. The task of full 3D human motion reconstruction from monocular underwater video remains largely underexplored, lacking both dedicated algorithms and large-scale annotated datasets.

This paper addresses the challenges of underwater HPS estimation by proposing an approach leveraging both 2D keypoint extraction and parametric model estimation. This approach is structured into two stages: preprocessing and optimization.

In the preprocessing stage, a Part Attention Regressor (PARE) [12] is employed to estimate SMPL human body parameters from individual frames within an underwater video sequence. This regressor can dynamically adjusts the importance of body parts, particularly those occluded, thereby enhancing the accuracy of SMPL parameter inference. This adaptability renders it particularly suitable for underwater scenarios, where the presence of water often obscures portions of the swimming body, leaving only the submerged parts identifiable.

Additionally, a 2D keypoint detector is employed to obtain skeletal keypoints from underwater imagery, serving as a supervision signal for subsequent optimization. Our approach leverages YOLO [13,14,15] for human bounding box detection and HRNet [16] as the regressor for 2D human keypoints. Notably, underwater environments present challenges such as lighting variations and water turbidity can degrade image quality and target visibility. HRNet [16], with its high-resolution network architecture, excels at feature extraction and reconstruction, enabling effective handling of low-quality and low-resolution underwater images.

The SMPL models obtained by PARE from the video sequence undergo further refinement in the optimization stage. An underwater variational autoencoder (UW-VAE) is pre-trained to learn the distribution of biomechanically plausible underwater human poses from data. Through this data-driven strategy, the learned prior implicitly corrects pose parameters distorted by refraction and occlusion, without requiring separate optical modeling of the water–air interface. Subsequently, an optimization algorithm is deployed, incorporating three constraints. Firstly, the reprojection of final SMPL models is aligned closer to the detected 2D keypoints. Secondly, the disparity between the adjusted SMPL model from UW-VAE and the original one is minimized. Lastly, ensuring the consistency and smoothness of SMPL models in the temporal domain is prioritized.

In addition to the inherent difficulties faced by computer vision-based methods in underwater sports and related domains, the absence of annotated datasets poses a significant challenge. However, recent research underscores the potential of synthetic data to effectively supplement or substitute real image data [17,18]. Building upon this insight, our paper introduces a pipeline that leverages the UW-VAE module to generate synthetic datasets specifically tailored for underwater sports, thereby addressing this critical data deficiency.

Through experimentation and evaluation, we demonstrate the effectiveness and applicability of our method in accurately estimating human body pose from single-view video sequences, thereby addressing a significant gap in current research and opening avenues for further exploration in underwater computer vision.

## 2. Related Work

This section reviews prior work most relevant to underwater monocular 3D HPS estimation. Since 2D keypoint detection serves as the foundation for monocular 3D reconstruction, we first briefly summarize existing terrestrial and aquatic approaches in this area. We then survey single-view 3D human reconstruction approaches, emphasizing that existing terrestrial methods cannot be directly transferred to underwater scenarios without substantial adaptation. Finally, we examine available underwater datasets and identify the scarcity of annotated 3D aquatic data. These gaps in 3D reconstruction methodology and data availability collectively motivate the methodology proposed in this paper. Recent work also explores AI-driven 3D perception for infrastructure resilience in extreme environments [19].

### 2.1. 2D Keypoint Detection

At present, methods of detecting 2D keypoints of the human body in terrestrial environments is relatively mature. Initially, human body keypoints estimation was directly treated as a classification or regression problem [20,21,22], yielding only modest accuracy, rendering them unsuitable for scenes with intricate backgrounds. With the advent of deep learning, DeepPose emerged as the pioneer, introducing a convolutional neural network (CNN) for the first time to address single-person pose estimation [23]. Subsequently, CPM [24] was introduced, employing heatmaps to determine keypoint positions, thus becoming the predominant paradigm for a considerable period [25]. In pursuit of a more lightweight model, the hourglass model was introduced refining the network structure based on CPM [24]. Concurrently, OpenPose leveraged Part Affinity Field (PAF) to assemble the human body, achieving remarkable results [26]. While many of these works prioritized encoder and context, sacrificing computational load by directly upscaling the image to enhance efficacy, HRNet subsequently emphasized the significance of spatial resolution [16].

Regarding keypoint detection in aquatic environments, early studies predominantly relied on manual labeling to extract 2D keypoints of human bodies. For instance, manual labeling was used in the photogrammetric assessment of swimmers’ 3D postures in a water tank as early as 1999. Traditional image feature treatments were also employed to extract underwater human keypoints for the analysis of large-scale data. Ferryanto et al. segmented swimming images by variable thresholds to obtain human body contours, and then extracted 2D keypoints using intersections of body parts [27]. Feng et al. used the canny operator to obtain the human body contour by leveraging the contrast between the swimmer’s body and the environment, and used the Hough method to identify and obtain 2D keypoints [28]. Recently, neural networks were introduced into the processing of underwater data with the development of deep learning. DeepLabCut was used to detect the 2D keypoints of the lower limbs in deep water running, which improved the accuracy and stability of detection [29]. Deep CNN was introduced to detect underwater human keypoints that adopted a coarse-to-fine strategy to extract keypoint information layer by layer through the CNN network [6]. SwimmerNet was proposed based on FCN (fully convolutional neural network) for underwater human keypoints extraction [1].

This paper adopts HRNet for underwater human keypoints extraction [16]. Compared with downsampling networks, HRNet considers multi-level scales to retain the information of the finest resolution layer, and then upsample through fused downsampling to obtain more contextual information and semantic layers. It effectively captures subtle variations in human poses and improves keypoint regression accuracy through information fusion from multiple branches. In challenging underwater environments with lighting variations and water turbidity, HRNet handles these complexities and delivers accurate pose estimates.

### 2.2. 3D Human Pose and Shape Estimation

Early methods reconstructed human geometric motion using contours or stereo vision [30,31], but they relied on dense-view data, consumed heavy load of computation, and often faced low robustness. After the proposal of statistical human models such as SMPL [11,32], methods have been proposed for single-view HPS estimation, which can be divided as optimization-based and regression-based according to different computation processes.

Optimization-based methods minimize an objective function to fit parametric body models to image observations. Initial efforts in this direction relied on contour cues to guide the fitting process [33]. The emergence of robust 2D keypoint detectors [24,25,26] subsequently enabled the use of detected 2D joint locations as reliable supervision signals, substantially improving reconstruction accuracy [34]. SMPLify uses an off-the-shelf 2D pose convolutional network to detect keypoints and perform gradient-based optimization, making the model better fit the observed image data [35]. Considering the mutual influence of the joints themselves, Guler et al. estimated the parameters of the human body model by extracting local features around human joints and generating features through a deconvolution network [36].

With the development of deep learning, regression-based methods [37,38,39] have gradually gained favor. ConvNet used the 2D keypoints and contours of the human body to predict parameters [40]. The NBF method trained its network end-to-end by combining 2D and 3D labels for robust parameter inference [41]. To solve the problem of joint drift caused by rotation of posture representation, DaNet used a position-assisted rotation feature refinement strategy to exploit the spatial relationship between body parts [42]. SPIN took the advantages of both optimization-based and regression-based methods, using regression estimation to obtain the initial parameters for the optimization, and then using the optimized results as strong supervision of the network [9]. To solve the occlusion problem, Part Attention Regressor (PARE) was proposed to set and supervise attention masks for body parts through partial segmentation, allowing the attention mechanism to utilize useful information from the body and surrounding pixels [12]. CLIFF [8] incorporated full-frame location information by considering both the bounding box position and the human subject’s focal length, enabling more accurate estimation of global body rotation and translation that are critical for downstream tasks. More recently, HMR 2.0 (4DHumans) [43] used transformers for robust tracking, SMPLer-X [44] scaled up expressive HPS with large-scale pretraining, and TokenHMR [45] introduced a tokenized pose representation for improved mesh recovery.

Beyond single-image methods, video-based approaches exploit temporal information to improve pose consistency across frames. VIBE [10] introduced a temporal encoder that processes sequences of SMPL parameters to produce temporally coherent outputs. TCMR [46] further improved temporal modeling by aggregating features across frames. GLAMR [47] addressed severe and long-term occlusion in dynamic-camera scenarios. For 2D-to-3D pose lifting from keypoint sequences, MotionBERT [48] proposed a unified pretraining paradigm that learns generalizable spatiotemporal representations from 2D keypoint sequences, achieving strong performance across multiple downstream human motion analysis tasks including 3D pose reconstruction. D3DP [49] employed a diffusion-based framework with multi-hypothesis aggregation to address the inherent ambiguity in monocular 3D pose estimation from 2D keypoint sequences. More recently, GLoT [50] proposed a global-to-local transformer for video-based HPS, and WHAM [51] reconstructed world-grounded humans with accurate 3D motion.

A complementary line of work explores generative pose priors to regularize HPS output. VPoser [52] pioneered the use of a VAE-based pose prior trained on a large corpus of SMPL poses, encoding a learned distribution of biomechanically plausible human body configurations. HuMoR [53] extended this idea to motion-level modeling with a conditional VAE that captures temporal pose dynamics. Pose-NDF [54] modeled the pose manifold as a neural distance field, offering an alternative to VAE-based representations. Our UW-VAE builds on this line of work by training a VAE-based prior specifically on underwater swimming data, enabling it to capture domain-specific pose distributions that generic land-based priors do not represent.

In summary, monocular 3D HPS estimation has achieved remarkable success in terrestrial environments through both optimization- and regression-based paradigms. However, these approaches implicitly rely on assumptions—such as clear imaging, reliable 2D keypoint detection, and abundant annotated training data—that are frequently violated underwater. Consequently, off-the-shelf terrestrial methods cannot be directly deployed in aquatic scenarios without substantial adaptation. To the best of our knowledge, dedicated monocular 3D human pose and shape estimation tailored for underwater conditions remains largely underexplored.

### 2.3. Underwater Human Motion Datasets

The scarcity of dedicated underwater 3D HPS methods is further compounded by the lack of annotated training data. While terrestrial datasets, such as CMU Panoptic [55], MPI-INF-3DHP [56], and MuPoTs-3D [57], are relatively abundant, aquatic data remain scarce due to challenging collection conditions. Camera deployment in aquatic environments is inherently difficult: above-water cameras are hindered by surface distortion and reflection, while underwater cameras face turbulence, limited visibility, and bubble occlusion. In addition, water depth and turbidity degrade image quality and reduce annotation accuracy.

More fundamentally, obtaining reliable 3D ground-truth annotations for underwater human poses is prohibitively challenging. Whether using marker-based or manual annotation, 3D pose labeling inherently requires a multi-view camera setup with precise inter-view calibration and temporal synchronization. In underwater environments, such setups require specialized hardware (e.g., Qualisys underwater cameras), complex multi-view calibration across the water–air interface, and correction for refraction-induced triangulation errors. Due to these technical and economic barriers, to the best of our knowledge, there is no publicly available real underwater dataset with 3D human pose ground truth.

Currently, the only publicly available real underwater dataset is Scylla [58], which provides contour annotations for swimmer silhouette extraction but lacks the 2D/3D keypoint and SMPL annotations necessary for 3D pose estimation. To bridge this gap, synthetic approaches have been explored. Generative datasets offer a practical alternative to real data collection: by learning the distribution of existing motion data, generative models can produce diverse samples with full annotations at low marginal cost [59]. Following this paradigm, Fiche et al. introduced SwimXYZ [60], a large-scale synthetic swimming dataset generated via GANimator, containing 11,520 videos with ground-truth 2D/3D keypoints and SMPL parameters across four stroke types.

With the development of deep learning and generative models, underwater datasets are expected to fill the data gap and further enrich the data resources in the field of underwater sports by combining real data and generated data.

## 3. Materials and Methods

**Figure 1:**
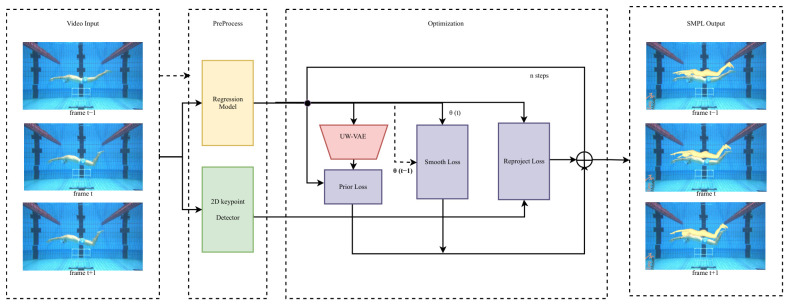
Overview of the UWPE pipeline, showing the two-stage workflow from input video to final 3D pose sequence via preprocessing and optimization.

In this paper, an underwater human pose estimation (UWPE) algorithm is proposed for realizing 3D human motion reconstruction through monocular RGB sequences in underwater scenes. As shown in Figure 1, the pipeline of UWPE is divided into two stages. In the preprocessing stage, a human motion regression model is used to predict the initial SMPL parameters for each frame in the video. In addition, the pixel coordinates of each 2D keypoint of the human body in each frame are obtained through a human 2D keypoint detector. In the optimization stage, the initial SMPL motion parameters are optimized in each iteration supervised by three constraints. An underwater variational autoencoder (UW-VAE) motion generation module is proposed to supervise the plausibility of the motion itself. Additionally, the predicted 2D keypoints help to supervise the reprojection of the motion in 2D images, and the motion needs to be kept smooth in the time domain.

### 3.1. Preprocess

**Initial SMPL parameter estimation.** PARE [12] is selected as the single-frame SMPL model regressor. This regressor can calculate the attentions of body parts from the corresponding 2D heatmaps, and use them as the weights for 3D feature map to regress the final SMPL model. When there are occlusions at some body parts, the network can implicitly eliminate their contributions to the final results. Due to the ubiquity of partial occlusion in underwater scenes, the advantages of PARE are particularly significant.

The regressed SMPL model can be expressed as Θ={θ,β,R,t} when a weak perspective camera model is adopted, where θ represents pose, and β represents shape. *R* and *t* are rotation and translation matrices, respectively.

**Figure 2:**
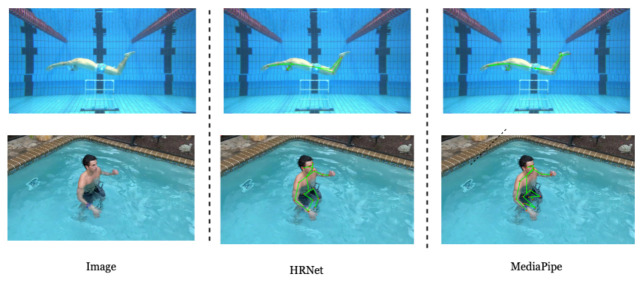
Qualitative comparison of 2D keypoint detection results from HRNet and MediaPipe.

**2D keypoint detection.** A pre-trained YOLO detector [14] is used to extract the bounding box of human body in each frame. Then, HRNet [61] is selected as the backbone for human keypoint detection. HRNet maintains high-resolution feature representations throughout the network, which is critical for keypoint localization in degraded underwater images affected by dynamic blur and water splash occlusion. In addition, HRNet supports model fine-tuning, enabling domain adaptation to underwater scenarios (Section 4). For reference, we also tested MediaPipe, a detector optimized for real-time on-device inference. As expected, HRNet produced more accurate keypoints on underwater images given its accuracy-oriented design, as illustrated in Figure 2. The predicted 2D keypoints are denoted as a set of $x_{i}\in R^{2\times K}$ where *i* indicates the i-th 2D keypoint.

### 3.2. Optimization

Underwater human motion data is often accompanied by various problems such as water wave occlusion, as well as water surface reflection and refraction [6]. The human motion of each frame of the video is distorted by refraction to a certain extent, and the SMPL parameter estimation can easily lead to unreasonable results due to the influence of occlusion.

In this stage, UW-VAE is proposed incorporating underwater motion prior information to the final reconstruction result, which can effectively avoid unreasonable output results caused by water wave occlusion, water surface reflection and refraction, etc. Then, three kinds of losses are utilized for each step of optimization to further enhance the robustness of inferred human motions.

**Figure 3:**
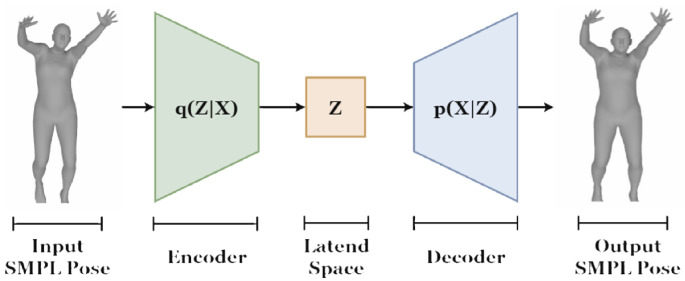
Architecture of the UW-VAE module. The encoder maps input SMPL pose parameters to a latent space; the decoder reconstructs the pose from the latent representation.

**Supervision of underwater motions.** UW-VAE is proposed as an action generation module by learning a low-dimensional latent space tailored for underwater motion scenes, where different latent vectors correspond to different human poses. UW-VAE adopts a data-driven strategy by learning the distribution of biomechanically plausible underwater poses from training data, and then the learned prior implicitly corrects pose parameters distorted by refraction and occlusion, without requiring separate optical modeling of camera intrinsics, water–air interface geometry, and refractive index, etc. UW-VAE aims to understand and represent the latent space of human poses, and thus generating lifelike pose samples while offering continuous pose manipulation and control. To achieve this, the network leverages a variational autoencoder (VAE) framework comprising an encoder [62] and decoder [63,64], as shown in Figure 3. The encoder transforms SMPL parameters of a single person into latent vectors via a neural network, encapsulating crucial features. Conversely, the decoder reconstructs these vectors into 3D human poses, striving for accurate reproduction. Detailed information of UW-VAE can be seen in Section 4.

As shown in Figure 3, the pre-trained UW-VAE is frozen and the initial pose parameters of SMPL model θ is taken as the input of UW-VAE to obtain a more reasonable pose $\theta^{\prime }$. The prior loss of the initial SMPL parameter is then obtained by calculating the mean square error (MSE) between θ and $\theta^{\prime }$, as illustrated in Equation (1).

(1) $$L_{\mathrm{prior}}=\sum_{i}{∥\theta_{i}-{\hat{\theta }}_{i}∥}_{2}$$

**Supervision of reprojection.** The predicted 2D keypoints in the preprocessing stage will be used as the supervision to calculate the reprojection loss $L_{\mathrm{reproj}}$ of the SMPL parameters, as shown in Equation (2).

(2) $$L_{\mathrm{reproj}}=\sum_{i}{∥\left(x_{i}-{\hat{x}}_{i}\right)∥}_{2}$$

where $x_{i}\in R^{2\times K}$ denotes the pixel coordinates of the i-th predicted 2D keypoints, and ${\hat{x}}_{i}$ denotes the pixel coordinates of the i-th reprojected 2D keypoints from SMPL model Θ. For a given Θ, the pixel coordinates of a reprojected 2D keypoint can be calculated as in Equation (3).

(3) $$\hat{x}=\Pi \left(RJ\left(\theta ,\beta \right)+t\right)$$

where J is the 3D joint regressor of SMPL model, and Π represents the orthogonal projection. Orthogonal projection is a practical choice, since full perspective projection with refraction correction requires camera intrinsics, water–air interface geometry, and refractive index that are typically unavailable for monocular in-the-wild underwater footage. The resulting domain shift is addressed by the data-driven implicit correction of the UW-VAE prior.

**Supervision of smoothness.** In the UWPE, the smoothness error in the video frame sequence consists of two components: skeleton length and pose velocity. The skeleton length is calculated by evaluating the change in skeletal length between the current frame and the previous frame using SMPL parameters. In human motion, the skeletal length should remain constant. The pose velocity is computed by measuring the rate of change between keypoints, which serves as an indicator of the degree of pose variation. Smaller velocity values indicate smoother pose transitions. To obtain more accurate and stable results, we optimize the motion smoothness loss. The formula for calculating the smoothness error is presented in Equation (4):

(4) $$L_{\mathrm{smooth}}=\sum_{t=2}^{T}\left({∥L\left(\theta_{t}\right)-L\left(\theta_{t-1}\right)∥}_{2}+{∥V\left(\theta_{t}\right)-V\left(\theta_{t-1}\right)∥}_{2}\right)$$

where *L* denotes the bone length and *V* represents the velocity of the human body’s keypoints. The two terms are summed with equal weights, as both operate on SMPL parameter-level qualities with comparable numerical scales. Note that the velocity penalty constrains the rate of change of pose velocity (i.e., acceleration), rather than velocity itself, so rapid movements such as freestyle kicking are not penalized as long as the motion remains temporally coherent.

**Optimization of SMPL parameters.** The shape parameters β are calculated by taking the average of the corresponding parameters of each frame in the entire underwater motion sequence. The pose parameters θ in all frames are optimized using the three above losses in Equations (1), (2), and (4). Reasonable weights are assigned to these three losses to obtain the final loss function, Equation (5).

(5) $$L_{\mathrm{total}}=\lambda_{1}L_{\mathrm{reproj}}+\lambda_{2}L_{\mathrm{smooth}}+\lambda_{3}L_{\mathrm{prior}}$$

Then, the posture estimation problem is to minimize the loss function, and gradually approach the optimal solution through an iterative optimization process. Gradient descent method is implemented for the optimization. When the change in θ before and after the iteration is less than the pre-set threshold, the optimization iteration is stopped and taken as the final result of human pose.

## 4. Underwater Dataset Generation

With the assistants of 3D asset editing tools such as Blender and Mixamo, a process for generating highly available simulation dataset is proposed utilizing the proposed UW-VAE module, as UW-VAE is able to quickly generate underwater motion data in response to the scarcity of 3D data in human underwater motion datasets. To a certain extent, the generated simulation data can replace the real image dataset, providing data support for subsequent human underwater motion capture tasks.

**Underwater human motion generation.** The proposed UW-VAE is capable of quickly and massively generating high-quality underwater moving human SMPL data. This enables versatile manipulation of poses through interpolation, sampling, and modification within the latent space, facilitating the generation of diverse pose samples.

The encoder and decoder modules of UW-VAE utilize fully connected layers with LeakyReLU activation functions, supplemented by Dropout layers to mitigate overfitting. The encoder maps the 72-dimensional SMPL pose parameters through hidden layers of size 512 and 256 to a 32-dimensional latent space; the decoder mirrors this architecture. During training, UW-VAE optimizes both encoder and decoder to minimize the standard VAE objective:

(6) $$L_{\mathrm{VAE}}={∥\theta -\hat{\theta }∥}_{2}+\beta \cdot D_{\mathrm{KL}}\left(q(z|\theta )\;∥\;N(0,I)\right)$$

where the first term is the reconstruction loss in SMPL parameter space, the second term is the KL divergence regularizing the latent distribution toward a standard Gaussian prior, and β controls the trade-off between reconstruction fidelity and latent space regularity.

The training data consists of two complementary sources. The first source is Subject 125 and Subject 126 from the CMU-Mocap dataset [65], whose motions were captured on dry land using a Vicon optical system (41 markers, 31 joints) with actors mimicking swimming strokes. The two subjects together provide 21 swimming-style sequences covering freestyle, backstroke, breaststroke, and butterfly. Despite the absence of real water resistance and buoyancy effects, these sequences provide high-precision kinematic templates of the target stroke categories, which serve as a clean structural prior for the VAE latent space. The second source is self-collected underwater motion data designed to inject domain-specific biomechanical characteristics. We gathered ∼2000 publicly available underwater images covering four stroke types across diverse pool environments. We then applied EasyMocap [66] for 2D keypoint detection and 3D SMPL parameter estimation, and manually screened the results to remove obviously incorrect poses. These lifted parameters, while less precise than optical motion capture, reflect the real underwater pose distribution that the CMU dry-land data cannot capture. The combined dataset is split into training and validation sets with an 80% and 20% ratio. Convergence typically occurs after approximately 200 training epochs.

By learning the plausible underwater human postures during training, UW-VAE is able to correct implausible per-pose estimates caused by water surface refraction or wave occlusion to a certain extent.

**Figure 4:**
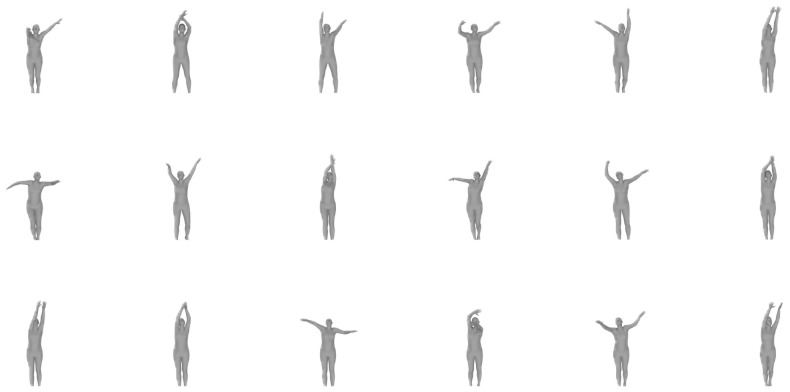
Sample underwater swimming poses generated by the UW-VAE module. Each figure represents a distinct SMPL body model rendered from randomly sampled latent codes.

Various SMPL parameters can be generated using pre-trained UW-VAE by randomly initializing the latent code in latent space and processing it through the decoder module. Figure 4 shows the effect of these parameters after visualization.

**Character rigging and scene production.** For generating a usable simulation dataset, the generated SMPL parameters are bound to a set of human body meshes selected from Mixamo. Then, the human body actions are produced with the help of Blender Add-on components. Each generated human body action is unique. In addition, the simulation scene is designed using Blender, which has a pool structure. The human meshes are inserted into the scene to finish the simulation data. Physical elements such as camera, lighting, and water refractive index are added to the scene to complete the production of the entire scene.

**Image rendering and ground-truth acquisition.** By randomly selecting a set of internal and external parameters of the camera, a 2D image is obtained by rendering the 3D simulation environment. Then, its corresponding SMPL parameters with camera parameters are provided as the label of the rendered image. Subsequently, The pixel coordinates of 2D human body keypoints can be calculated by Equation (3). Consequently, each data in the final dataset comprises an image with its ground-truth labels including SMPL and camera parameters, and 2D keypoint coordinates.

## 5. Experiment and Applications

In this section, we evaluate UWPE on the SwimXYZ dataset [60], a large-scale synthetic benchmark for underwater swimming motions. We first introduce the dataset and the evaluation metrics used for quantitative comparison. We then compare UWPE against several state-of-the-art monocular 3D HPS methods, including PARE [12], CLIFF [8], D3DP [49], and MotionBERT [48], across four swimming categories. Extensive ablation studies are conducted to investigate the contribution of the UW-VAE module and the influence of individual loss terms. Finally, we validate the utility of the synthetic data generated by UW-VAE by fine-tuning a 2D keypoint detector.

### 5.1. Experimental Setup

**Implementation.** All experiments are conducted on a workstation equipped with an NVIDIA RTX 3090 GPU. The UWPE pipeline is implemented in PyTorch. For the preprocessing stage, we adopt the publicly available pre-trained PARE model for SMPL parameter regression and HRNet for 2D human keypoint detection. The UW-VAE module is trained from scratch using the Adam optimizer. Its encoder and decoder are composed of fully connected layers with LeakyReLU activation functions and Dropout layers for regularization. The training data for UW-VAE comprises Subject 125 and 126 from the CMU-Mocap dataset [65], together with self-collected underwater motion data. Convergence is typically reached after approximately 200 epochs.

**Dataset.** SwimXYZ [60] is a synthetic dataset dedicated to swimming, with 11,520 synthesized monocular videos annotated with ground-truth 2D and 3D joints, as well as 240 swimming motion sequences in SMPL parameter format. A total of 3.4 million frames are included in SwimXYZ, varying in camera angles, subject and water appearance, lighting, and motion. Data was generated using GANimator with four types of swimming movements: backstroke, breaststroke, butterfly stroke, and freestyle. No camera parameters are provided in SwimXYZ.

**Evaluation Metrics.** Since SwimXYZ does not provide camera intrinsic or extrinsic parameters, absolute scale alignment is infeasible; therefore, we adopt Procrustes Mean Per Joint Position Error (PMPJPE), which performs Procrustes Analysis to align the estimated pose with the ground-truth pose in scale, rotation, and translation before calculating the mean joint position error. This alignment helps to mitigate discrepancies due to differences in pose orientation or scale between the estimated and ground-truth poses. The closer the predicted 3D joints are to the true value, the smaller the PMPJPE value is.

In addition, we report Percentage of Correct Keypoints (PCK), a widely used evaluation metric in the field of 2D human pose estimation. It measures the percentage of keypoints that fall within a certain threshold distance from their ground-truth positions. Typically, the threshold distance is defined as a certain percentage of the length of the person’s body part. In this paper, we use the length of the bounding box’s diagonal as the normalization factor, a common alternative that avoids dependence on individual body part dimensions. A higher PCK value indicates better performance, as it means that a larger proportion of keypoints are accurately predicted by the model. Common thresholds include PCK@0.2 and PCK@0.5, which correspond to 20% and 50% of the body part length, respectively. PCK@0.2 requires higher precision, while PCK@0.5 allows for more deviation.

### 5.2. Experimental Results

The experimental results are compared with the SOTA single-view 3D human pose estimation algorithms in non-special scenarios, specifically, CLIFF [8] and PARE [12], as research in the field of underwater human motion estimation is still in its infancy. An ablation study is also conducted to analyze the influence with and without the UW-VAE module on the UWPE algorithm in various indicators.

**Figure 5:**
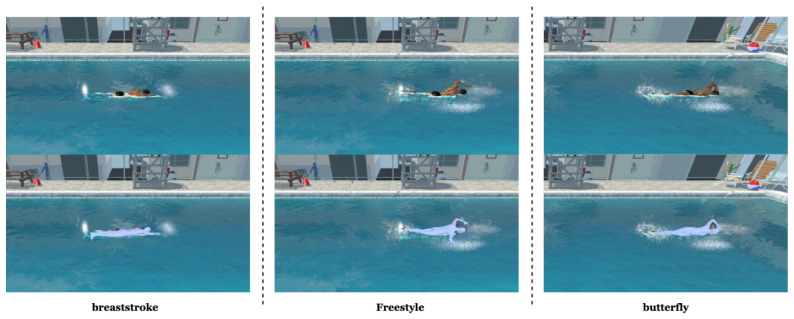
Qualitative results of UWPE on SwimXYZ across different stroke types, including breaststroke, freestyle and butterfly.

**Comparison Experiment.** Quantitative comparisons are conducted on the four sports categories on SwimXYZ: backstroke, breaststroke, butterfly stroke, and freestyle. Some visualization results are shown in Figure 5. The comparison results using PMPJPE as the indicator are shown in Table 1, and the ones using PCK@0.2 and PCK@0.5 are shown in Table 2. All baseline methods are evaluated using their publicly available pre-trained checkpoints without further fine-tuning on SwimXYZ. PARE [12] and CLIFF [8] take RGB images as input and directly regress SMPL parameters. D3DP [49] and MotionBERT [48] take 2D keypoint sequences as input and reconstruct 3D poses. All methods are evaluated under the same PMPJPE and PCK metrics described in Section 5.1. Since our UWPE also uses only publicly available pre-trained models for preprocessing and does not benefit from SwimXYZ-specific training of these components, the comparison is fair and conservative.

As shown in Table 1 and Table 2, our method achieves the lowest PMPJPE for three of the four stroke categories and the highest overall PCK, while MotionBERT obtains a slightly better PMPJPE for breaststroke. The overall advantage of UWPE can be attributed to the optimization process introduced in Section 3.2, which better utilizes constraints and prior knowledge to obtain more accurate pose estimates. The iterative refinement during optimization also helps correct incomplete or noisy input data, making the method robust to underwater challenges such as motion blur and occlusion.

**Ablation Experiment.** In the ablation experiment, the effectiveness of the UW-VAE module is investigated by comparing the performance of our algorithm with and without UW-VAE. The algorithm without UW-VAE indirectly eliminates UW-VAE by setting the weight of the prior loss to zero. The comparison results are shown in Table 3, which indicate that the algorithm with UW-VAE shows clear advantages in performance. The algorithm with UW-VAE achieved an accuracy improvement of approximately 10% in PMPJPE, and an improvement of about 5% in the PCK indicator. This advantage can be attributed to the introduction of the UW-VAE module, which makes the estimation results more consistent with human motion characteristics by reasonably limiting and constraining underwater motion postures.

**Figure 6:**
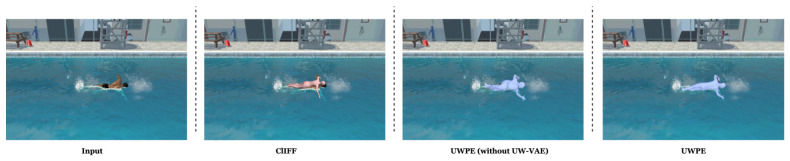
Visual comparison of 3D pose reconstruction on SwimXYZ. UWPE with the UW-VAE prior produces anatomically plausible poses, while removing the prior leads to unnatural body configurations despite reasonable 2D keypoint alignment.

Qualitative results on some data in SwimXYZ are visualized as shown in Figure 6, and the results of our algorithm are more consistent with the human body structure, which further verifies the key role of the UW-VAE module in underwater posture estimation. By introducing reasonable constraints and restrictions, the UW-VAE module can effectively improve the accuracy and reliability of posture estimation, making the posture results closer to the real human motion characteristics.

To investigate the influence of individual weights within the loss function, we conducted a series of ablation studies by adjusting the relative magnitude of each weight and observing its impact on performance.

We first varied the weight $\lambda_{1}$ of the $L_{\mathrm{reproj}}$ and documented the corresponding changes in the PMPJPE of the model. The results indicated that when $\lambda_{1}$ was set too low, the model tended to neglect the accuracy of the 2D keypoint detection, leading to a substantial increase in PMPJPE. Conversely, when $\lambda_{1}$ was excessively high, the model became overly reliant on 2D keypoints, disregarding the intrinsic plausibility of the 3D human model.

Next, we performed a similar ablation study on the weight $\lambda_{2}$ of the $L_{\mathrm{smooth}}$. Adjustments to $\lambda_{2}$ revealed an optimal weight range for maintaining the coherence of pose transitions. A weight that was too low resulted in abrupt pose changes, while an excessively high weight led to overly smooth transitions, sacrificing precision at certain keypoints.

Lastly, we adjusted the weight $\lambda_{3}$ of the $L_{\mathrm{prior}}$. The prior loss helps the model generate physically plausible human poses. The results highlighted that the weight $\lambda_{3}$ is particularly crucial for the model’s performance in complex underwater environments, where a well-chosen $\lambda_{3}$ could effectively reduce abnormal pose estimations caused by the underwater environment.

**Figure 7:**
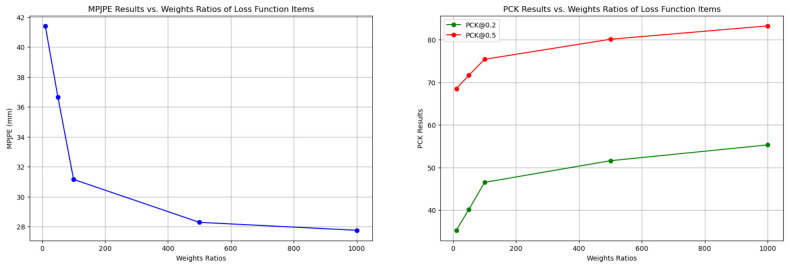
Sensitivity of PMPJPE and PCK to the ratio between reprojection weight and prior weight.

The comprehensive results from these ablation studies revealed a delicate balance between the weights of different components in the loss function. Through a grid search over the weight space, we identified the optimal combination of $\lambda_{1}$, $\lambda_{2}$, and $\lambda_{3}$ for $L_{\mathrm{reproj}}$, $L_{\mathrm{smooth}}$ and $L_{\mathrm{prior}}$, respectively. An illustrative example of this grid search is shown in Figure 7. This ablation study not only substantiates the efficacy of our approach but also provides valuable insights for adjusting the weights of the loss function in similar tasks.

### 5.3. Quality of Our Simulation Dataset

**Figure 8:**
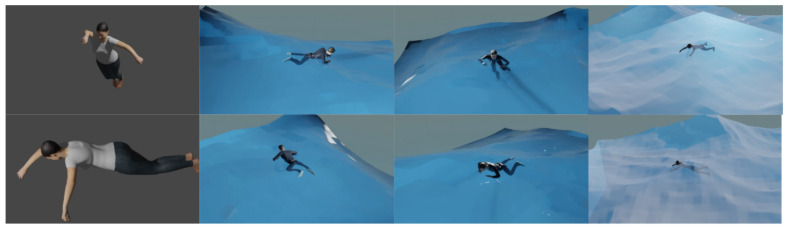
Partial examples of the underwater human motion simulation dataset generated by the UW-VAE module.

The pipeline introduced in Section 4 is used for the generation of our simulation dataset which contains about 2000 images. The scene is rendered from eight different perspectives by setting random lighting intensity for real environment simulation using Blender. In addition, HRNet is fine-tuned using the generated dataset. Figure 8 shows some of the generated images, which are used for fine-tuning.

**Figure 9:**
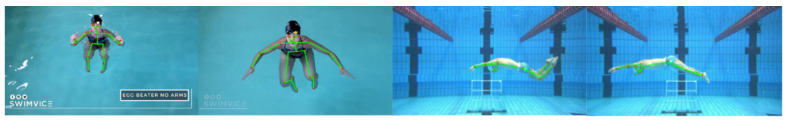
2D keypoint detection results by the fine-tuned HRNet. Left is on synthetic images, and right is on real underwater images.

We evaluate the transfer capability of the fine-tuned HRNet detector on real underwater images collected from publicly available online swimming videos and photographs, covering four stroke types across diverse pool environments. We manually annotated 2D keypoints on ∼500 images in COCO format. The quantitative results are shown in Table 4 with some qualitative results shown in Figure 9. The results indicate that fine-tuning on our synthetic data improves 2D keypoint accuracy on real underwater images.

Using GAN [67] as the generation network as in SwimXYZ [60] faces some limitations. Multiple independent generators and discriminators need to be trained for different motion categories since the training method of GAN is carried out in a competitive and adversarial manner. This multi-generator structure increases the model complexity and requires more training time and computing resources. In contrast, our UW-VAE model adopts the structure of a variational autoencoder (VAE), which only requires one encoder and one decoder to generate different types of underwater human motion. This simplified model structure not only reduces the complexity of training and inference, but also improves the efficiency of generating underwater human motion.

In addition, our UW-VAE model also shows higher diversity in generating underwater human body movements. By learning the distribution of the latent space, UW-VAE can generate richer and more diverse action samples, thereby simulating a wider range of underwater motion scenarios. Compared with the GANimator, UW-VAE has a greater advantage in generating diverse actions.

## 6. Conclusions

This paper proposes an underwater human pose estimation (UWPE) algorithm, which aims to achieve realistic underwater human motion reconstruction through monocular RGB sequences. In this algorithm, the initial human body SMPL parameters and 2D keypoint coordinates are obtained in the preprocessing stage. Then, the SMPL action parameters are optimized for solving problems in underwater scenes such as occlusion, reflection, etc. The core innovation of the algorithm is the proposed UW-VAE action generation module, which adds underwater motion prior information to the reconstruction results and avoids unreasonable output due to problems such as water wave occlusion. In addition, UWPE uses a variety of loss functions, including reprojection loss, action prior loss and action smoothing loss, to obtain accurate SMPL parameters through gradient descent optimization. Experimental results on the SwimXYZ synthetic benchmark demonstrate that UWPE achieves substantial improvements over traditional land-based methods. Furthermore, the UW-VAE action generation module is able to quickly generate a large amount of high-quality underwater human motion data, and thus can be used for addressing scarcity of underwater data and providing data support for related tasks.

Despite the promising performance of UWPE, several limitations remain. First, quantitative 3D evaluation is currently limited to synthetic benchmarks, as no public real underwater 3D ground-truth dataset exists. The synthetic images cannot fully capture real underwater phenomena such as bubbles, splash, and variable visibility. Future work will also extend the method to diverse underwater activities beyond swimming (e.g., diving, underwater rescue, synchronized swimming) to improve generalization. Second, this work focuses on single-person scenes, whereas real underwater environments may involve multi-person interaction, obstacles, and dynamic surface interference. Extending the method to multi-person settings and introducing domain-adaptive modules is an important future direction. Finally, the current pipeline is validated on a desktop GPU; model compression and lightweight design will be explored to enable deployment on portable underwater devices for real-time analysis.

From a practical perspective, the temporally consistent SMPL parameters produced by UWPE can serve as input to downstream biomechanical analysis workflows. Potential applications include joint-angle trajectory extraction across stroke cycles, left–right asymmetry assessment, and longitudinal technique monitoring across training sessions. The current pipeline operates offline: a coach records a swimming video using a consumer camera, and the system processes it afterward to produce 3D pose sequences. Real-time deployment would require GPU optimization of the iterative optimization loop, which we identify as future engineering work.

In general, underwater human pose estimation is a very challenging interdisciplinary problem, which requires knowledge and technology from multiple fields such as computer vision, pattern recognition, and physical modeling. Continuous innovation and hard work should be made in this field to make great progress and make important contributions to underwater sports science research and related applications. 

## Figures and Tables

**Table 1:** UWPE outperforms the other two regression-based 3D human pose estimation algorithms in terms of PMPJPE metric (mm).

Methods	Freestyle	Butterfly	Breaststroke	Backstroke
PARE [12]	62.52	46.32	33.57	32.16
CLIFF [8]	59.17	48.85	29.09	32.80
D3DP [49]	41.12	33.50	27.32	30.16
MotionBERT [48]	46.38	26.56	**21.46**	29.24
UWPE(Ours)	**36.88**	**25.34**	22.37	**28.58**

Best results are in bold.

**Table 2:** Performance of PCK metrics on the SwimXYZ dataset.

Methods	PCK@0.2	PCK@0.5
PARE [12]	41.50	72.55
CLIFF [8]	44.00	70.67
D3DP [49]	50.75	77.57
MotionBERT [48]	47.55	76.98
UWPE(Ours)	**51.60**	**80.13**

Best results are in bold.

**Table 3:** Performance of the UWPE on PMPJPE (mm) and PCK metrics with and without the UW-VAE Module.

Methods	PMPJPE	PCK@0.2	PCK@0.5
without uw-vae	31.26	48.67	76.93
final	**28.29**	**51.60**	**80.13**

Best results are in bold.

**Table 4:** PCK evaluation of HRNet fine-tuning on real underwater images.

	PCK@0.1	PCK@0.2	PCK@0.5
HRNet [61]	13.26	23.64	45.34
HRNet-finetuned	**30.26**	**58.60**	**80.13**

Best results are in bold.

## Data Availability

Data available on reasonable request.
